# Supplementary material for: SH3BP2 Silencing Increases miRNAs Targeting ETV1 and Microphthalmia-Associated Transcription Factor, Decreasing the Proliferation of Gastrointestinal Stromal Tumors
Source: Cancers (Basel). 2022 Dec 15;14(24):6198. doi: 10.3390/cancers14246198 (PMC9777313; doi:10.3390/cancers14246198)
Supplement: Supplementary file 1 [file cancers-14-06198-s001.zip › cancers-2058158-supplementary material S1-WB.pdf]

SH3BP2 silencing increases miRNAs targeting ETV1 and microphthalmia-associated transcription factor, decreasing the proliferation of gastrointestinal stromal tumors.

Elizabeth Proaño-Pérez, Eva Serrano-Candelas, Cindy Mancia, Arnau Navinés-Ferrer, Mario Guerrero and Margarita Martín.

UNCUT BLOTS

Figure 1.

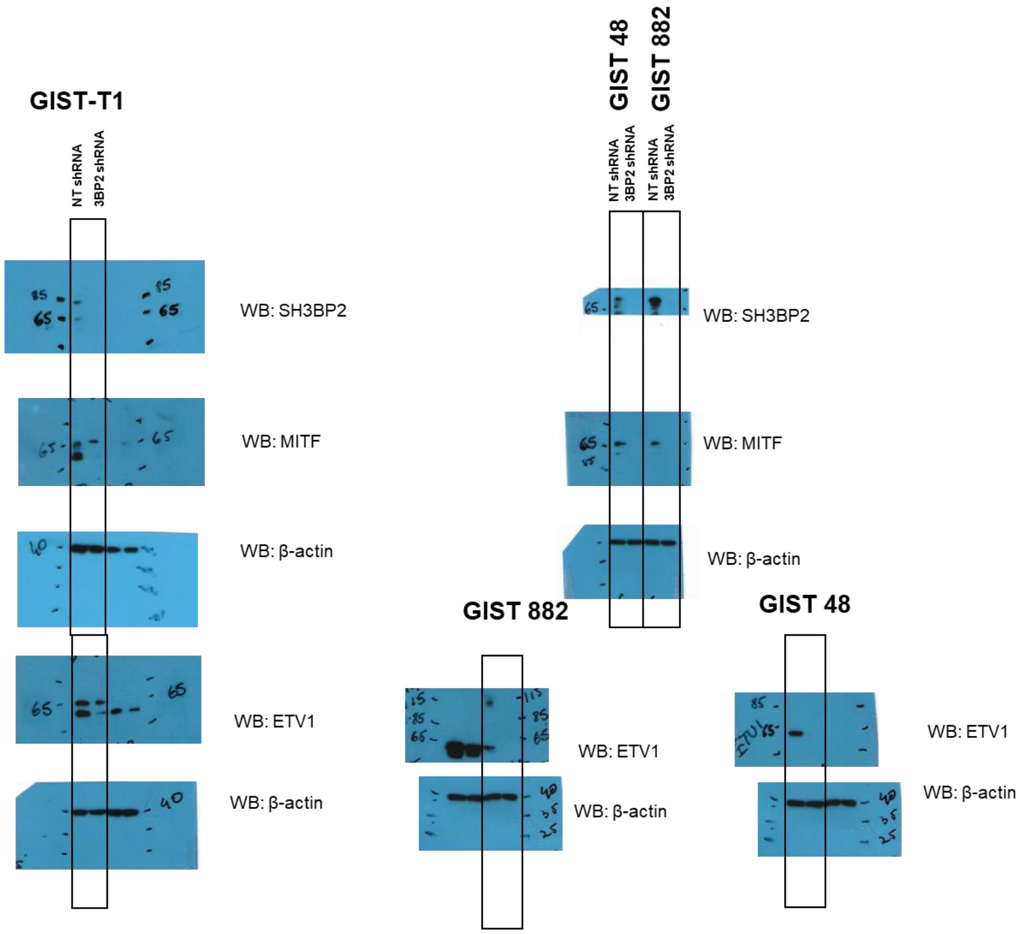

Figure 1: Western blot images with molecular weight for MITF, ETV1, and SH3BP2. β-actin was used as a loading control, bands shown in Figure 1. The black rectangle indicates the area demonstrated in Figure 1.

Figure 4A.

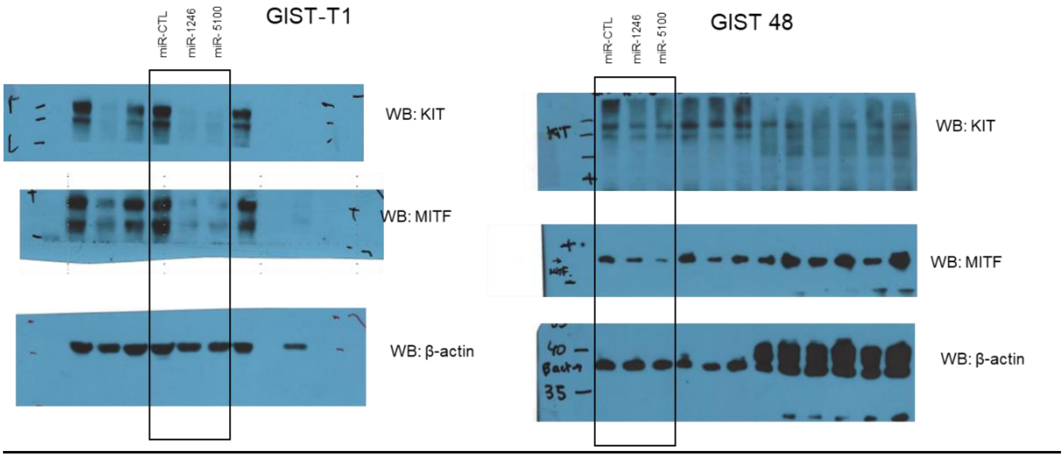

Figure 4A. Western blot images with molecular weight for KIT, MITF, and the respective β-actin control bands shown in Figure 4A. The black rectangle indicates the area shown in Figure 4A.

Figure 4B.

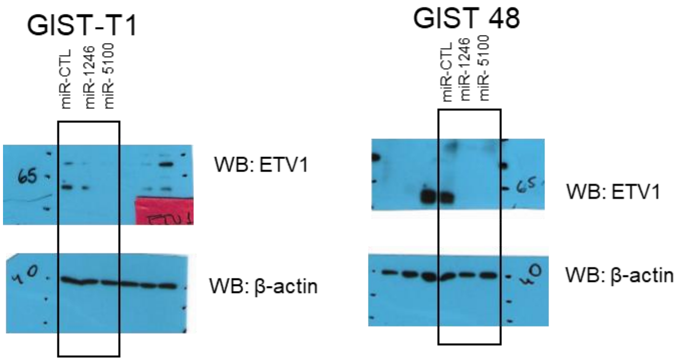

Figure 4B. Western blot images with molecular weight for ETV1, and β-actin control bands shown in Figure 4B. The black rectangle indicates the area shown in Figure 4B.

Figure 5A.

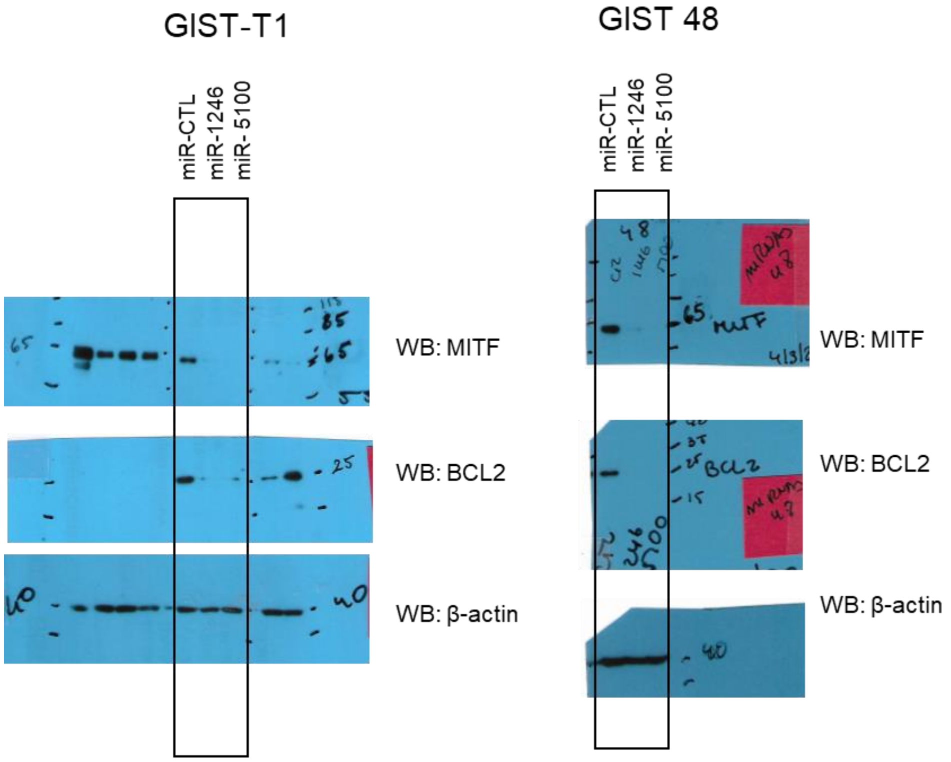

Figure 5A. Western blot images with molecular weight for MITF, BCL2, and β-actin control bands demonstrated in Figure 5A. The black rectangle indicates the area shown in Figure 5A.

Figure 6A.

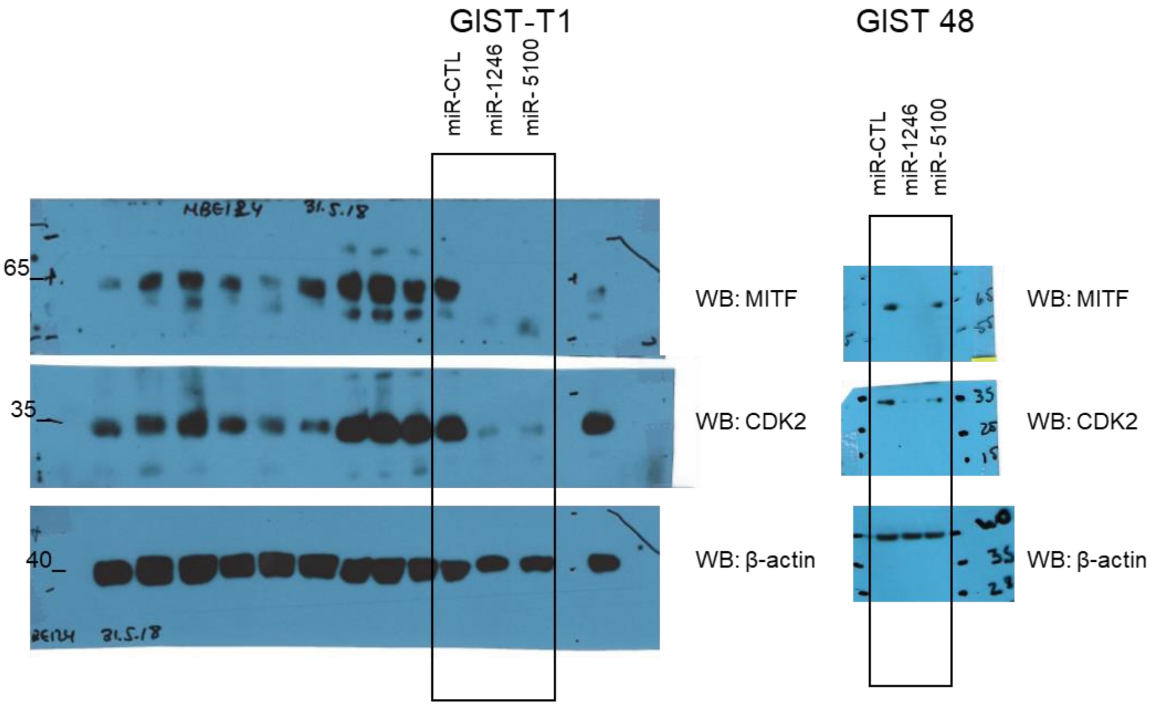

Figure 6A. Western blot images with molecular weight for MITF, CDK2, and β-actin control bands shown in Figure 6A. The black rectangle indicates the area indicated in Figure 6A.

Figure 7.

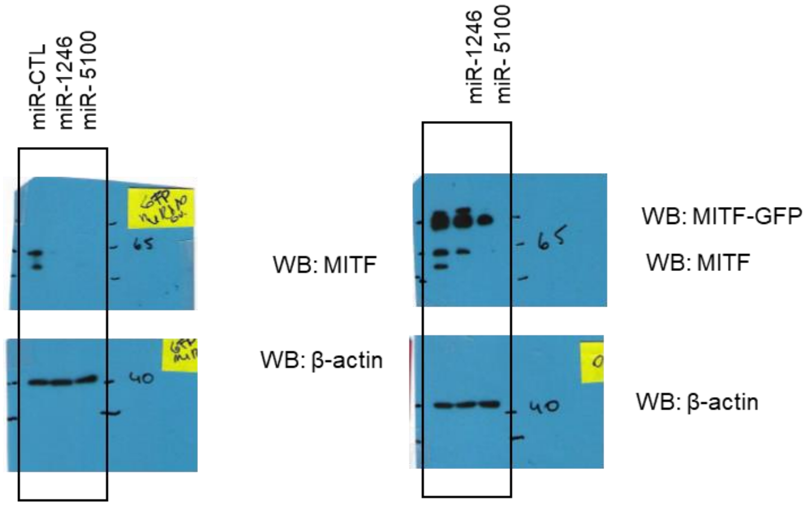

Figure 7. Western blot images with molecular weight for MITF-GFP and MITF (endogenous), and β-actin control bands shown in Figure 7. The black rectangle indicates the area indicated in Figure 7.
