# Supplementary material for: SH3BP2 Silencing Increases miRNAs Targeting ETV1 and Microphthalmia-Associated Transcription Factor, Decreasing the Proliferation of Gastrointestinal Stromal Tumors
Source: Cancers (Basel). 2022 Dec 15;14(24):6198. doi: 10.3390/cancers14246198 (PMC9777313; doi:10.3390/cancers14246198)
Supplement: Supplementary file 1 [file cancers-14-06198-s001.zip › cancers-2058158-supplementary Tables and Figures.pdf]

SupplementaryMaterials

SH3BP2 silencing increases miRNAs that target ETV1 and Microphthalmia-associated transcription factor, decreasing Gastrointestinal stromal tumors proliferation.

Elizabeth Proaño-Pérez, Eva Serrano-Candelas, Cindy Mancia, Arnau Navinés-Ferrer, Mario Guerrero and Margarita Martín.

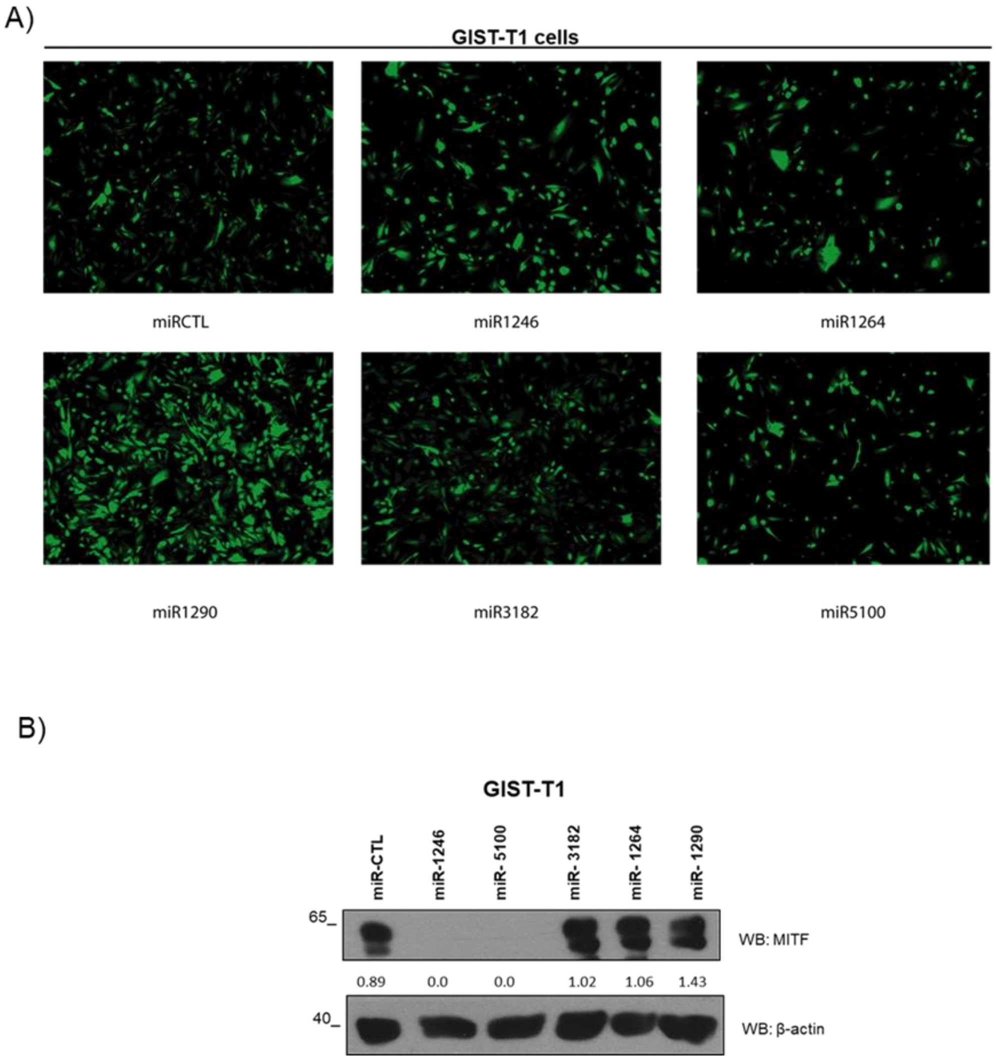

Figure S1. MiRNAs validation by western blot. Selected miRNAs-GFP were overexpressed in GIST-T1 cells. Levels of transfection were checked by fluorescence microscopy (A). MiRNAs transfected cells were lysed, and a western blot was performed. MITF was detected with a specific antibody. β-actin was used as a loading control. Numbers are the ratio MITF/β-actin.

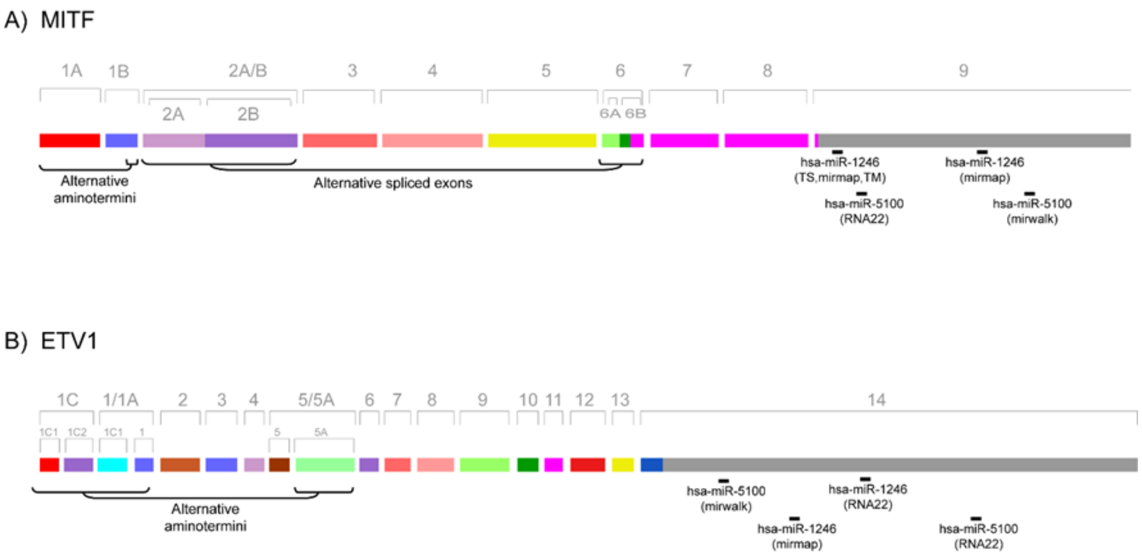

Figure S2. MiRNA sequences target on 3'UTR of MITF (A) and ETV1 (B) genes. The predicted miRNAs sequences targeting 3'UTR of MITF and ETV1 have been predicted using different software (TS (targetscan), mirmap, RNA22, mirkwalk, and TM (targetminer) software.

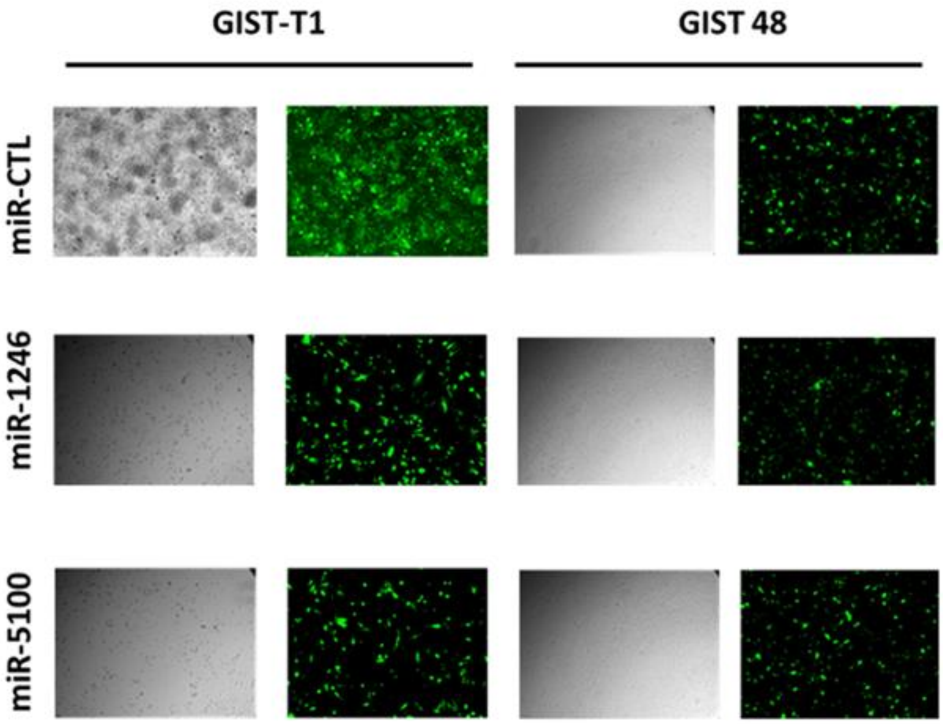

Figure S3. MiR-GFP expression in GIST cells. GFP expression was checked by fluorescence microscopy to assess lentiviral transduction for all the miR-GFP constructs: miR-CTL, miR-5100, and miR-1246.

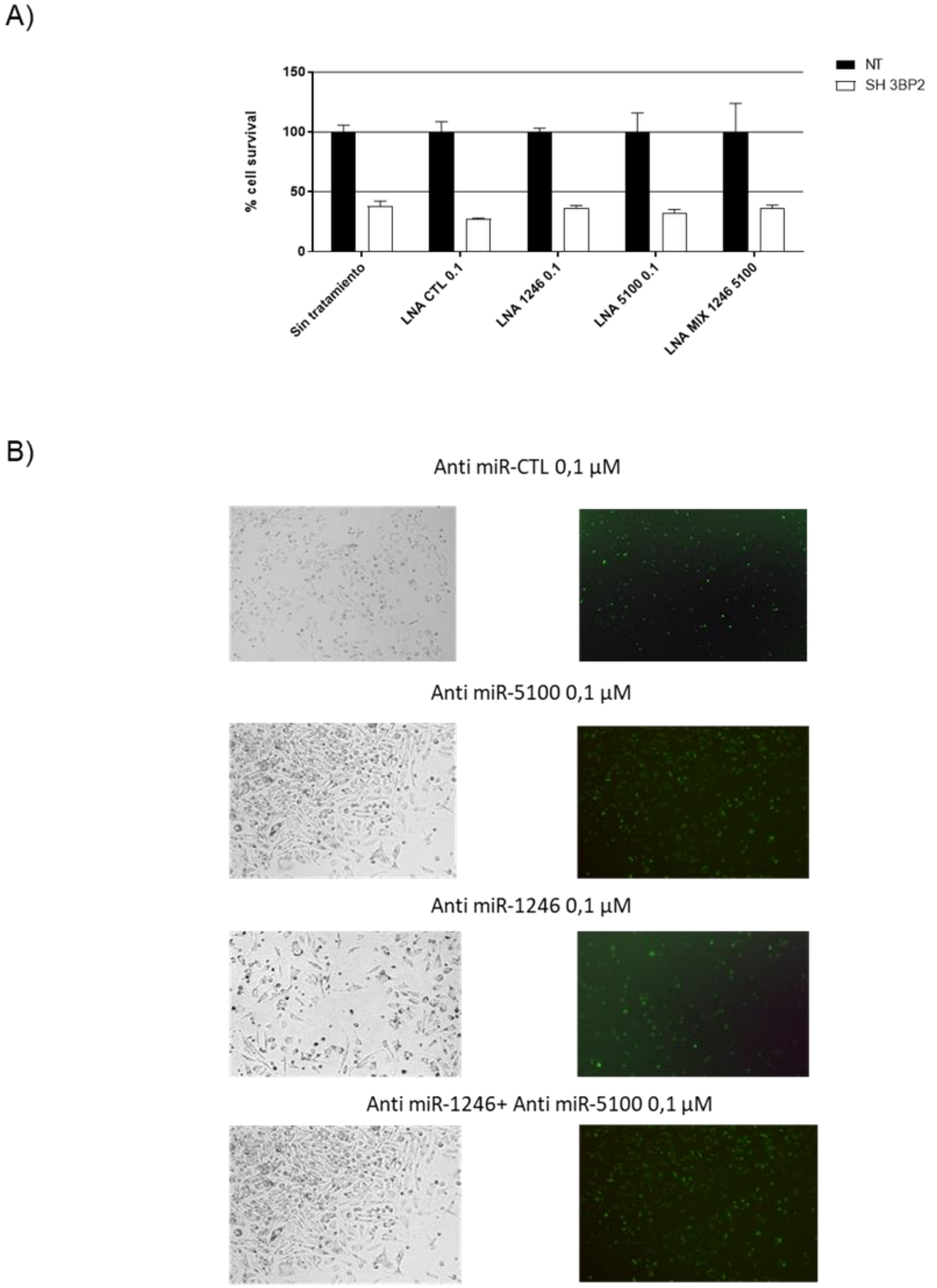

Figure S4. Anti-miRNA treatment 1246 and 5100 in GIST T1. LNA treatment was performed on SH3BP2 silenced cells. (A) Viability was measured by crystal violet staining at 4 days after lentiviral transduction. (B) Fluorescence FAN LNA miR-CTL, miR-5100, and miR-1246 were measured by fluorescence microscopy 24h after LNA treatment and monitored along the treatment.

Table S1. Prediction of miRNAs that target MITF by different databases.

Database	hsa-miR-1246	hsa-miR-1264	hsa-miR-1290	hsa-miR-3182	hsa-miR-5100
TargetScan7	✓	✓	✓	✓	✓
microT CDS	✓	✓	×	×	×
mirwalk 2.0	×	✓	×	✓	✓
mirDIP	✓	✓	×	×	×
mirmap	✓	✓	✓	×	×
RNA22	×	×	×	×	✓
Targetminer	✓	✓	×	×	✓

Target prediction shows if MITF is a target (√) or not (×) of the five most upregulated miRNAs in SH3BP2-silenced GIST882 and GIST48 found in a miRNA microarray.

Table S2. Prediction of miRNAs that target ETV1 by different databases.

Database	hsa-miR-1246	hsa-miR-1264	hsa-miR-1290	hsa-miR-3182	hsa-miR-5100
TargetScan7	×	×	✓	✓	×
microT CDS	×	×	×	✓	×
mirwalk 2.0	✓	✓	×	×	✓
mirDIP	×	✓	✓	✓	×
Mirmap	✓	×	✓	✓	×
RNA22	✓	✓	✓	✓	✓
Targetminer	✓	✓	✓	✓	✓

Target prediction shows if ETV1 is a target (√) or not (×) of the five most upregulated miRNAs in SH3BP2-silenced GIST882 and GIST48 found in a miRNA microarray.
